# Intestinal Subepithelial Myofibroblasts Support *in vitro* and *in vivo* Growth of Human Small Intestinal Epithelium

**DOI:** 10.1371/journal.pone.0026898

**Published:** 2011-11-17

**Authors:** Nicholas Lahar, Nan Ye Lei, Jiafang Wang, Ziyad Jabaji, Stephaine C. Tung, Vaidehi Joshi, Michael Lewis, Matthias Stelzner, Martín G. Martín, James C. Y. Dunn

**Affiliations:** 1 Division of Gastroenterology and Nutrition, Department of Surgery, David Geffen School of Medicine, University of California, Los Angeles, California, United States of America; 2 Division of Gastroenterology and Nutrition, Department of Bioengineering, David Geffen School of Medicine, University of California, Los Angeles, California, United States of America; 3 Division of Gastroenterology and Nutrition, Department of Pediatrics, David Geffen School of Medicine, University of California, Los Angeles, California, United States of America; 4 Department of Pathology, Veterans Affairs Greater Los Angeles Healthcare System, University of California, Los Angeles, California, United States of America; The University of Texas M.D Anderson Cancer Center, United States of America

## Abstract

The intestinal crypt-niche interaction is thought to be essential to the function, maintenance, and proliferation of progenitor stem cells found at the bases of intestinal crypts. These stem cells are constantly renewing the intestinal epithelium by sending differentiated cells from the base of the crypts of Lieberkühn to the villus tips where they slough off into the intestinal lumen. The intestinal niche consists of various cell types, extracellular matrix, and growth factors and surrounds the intestinal progenitor cells. There have recently been advances in the understanding of the interactions that regulate the behavior of the intestinal epithelium and there is great interest in methods for isolating and expanding viable intestinal epithelium. However, there is no method to maintain primary human small intestinal epithelium in culture over a prolonged period of time. Similarly no method has been published that describes isolation and support of human intestinal epithelium in an *in vivo* model. We describe a technique to isolate and maintain human small intestinal epithelium *in vitro* from surgical specimens. We also describe a novel method to maintain human intestinal epithelium subcutaneously in a mouse model for a prolonged period of time. Our methods require various growth factors and the intimate interaction between intestinal sub-epithelial myofibroblasts (ISEMFs) and the intestinal epithelial cells to support the epithelial *in vitro* and *in vivo* growth. Absence of these myofibroblasts precluded successful maintenance of epithelial cell formation and proliferation beyond just a few days, even in the presence of supportive growth factors. We believe that the methods described here can be used to explore the molecular basis of human intestinal stem cell support, maintenance, and growth.

## Introduction

The intestinal epithelium is composed of a perpetually dividing epithelium composed of five primary cell types: the common absorptive enterocyte, the enteroendocrine cell, the mucous secreting goblet cell, the tuft cell, and the Paneth cell[1], [2]. These cells are continuously being renewed at the base of the crypts of Lieberkühn where the intestinal stem cells reside [3]. These progenitors differentiate on their journey up the crypt to the villus tip, and these crypt-villus units comprise the functional element of the intestinal epithelium[1], [4].

Intestinal stem cells are of great interest for their potential clinical applications[4]. Significant advances have recently been made in the understanding of the intimate interaction between these stem cells, which are found at the base of intestinal crypts, and the surrounding milieu[5], [6].

Of particular interest among the factors that play a role in the stem cell niche are the intestinal subepithelial myofibroblasts (ISEMFs). These cells are located in the lamina propria in close proximity to the crypt cells[6]. ISEMFs have qualities of both smooth muscle cells and fibroblasts[7]. They interact via various conserved intracellular pathways such as Wnt, Bmp, and Notch to regulate stem cell behavior, likely via both direct contact and paracrine modalities [2], [8], [9], [10]. However, ISEMFs are not the only cells that have been shown to have supportive and regulatory effects upon crypt stem cells. Recently, Paneth cells have been implicated in the maintenance of intestinal stem cells and likely interact via pathways similar to ISEMFs[11]. The alternating pattern of Paneth cell and crypt stem cells at the crypt base speak to the intimate contact of these cell types, much like that between the ISEMFs and the crypt stem cells [5]. Indeed, Lgr5+ stem cells grown *in vitro* in the presence of Paneth cells were shown to form intestinal epithelial cell structures in a significantly higher number than for stem cells cultured alone [11]. Additionally, myofibroblasts are just one of a variety of mesenchymal cells found in the crypt-villus niche. Recent studies show that there are several different variably smooth muscle actin positive mesenchymal cells in the lamina propria with a variety of other cell surface markers that may also contribute to the functionality of the intestinal epithelium [12]. Although ISEMFs have been frequently associated with regulation of intestinal epithelium, clearly multiple factors and cell types play a role in intestinal stem cell regulation. ISEMFs likely play other supportive roles; subepithelial myofibroblast migration may promote epithelial regrowth and enhance barrier function during times of injury or stress [13]. Electron microscopy has demonstrated migration of myofibroblast through basement membrane pores following the loss of overlying epithelium [14].

In this study we demonstrate the ability of both mouse and human ISEMFs to support the growth, differentiation, and expansion of human intestinal epithelium from previously isolated human crypts. We demonstrate that myofibroblasts are required to maintain human epithelial cells on a long-term basis in a culture environment. We also demonstrate that mouse myofibroblasts can maintain human epithelial cell clusters subcutaneously *in vivo*. These cultured human epithelial cells exhibit immunohistochemical markers for complete, mature intestinal epithelium.

## Results

### Evaluation of murine ISEMFs

**Figure 1:**
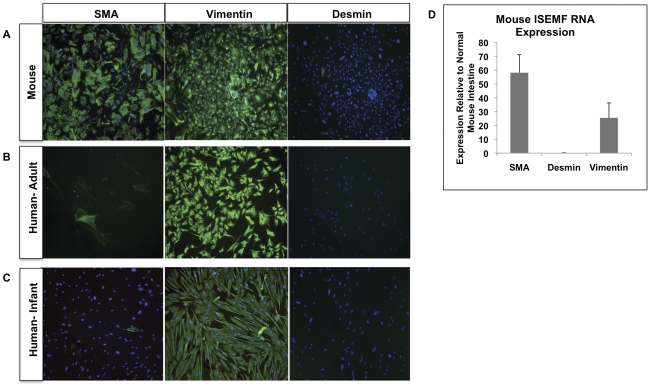
Characterization of Mouse and Human Myofibroblasts. (A) C57 BL/6 murine myofibroblasts plated in plastic culture dishes for four days. Using immunofluorescence, cells stained characteristically for intestinal myofibroblasts with positive SMA and vimentin staining and negative desmin staining. The blue pseudocolor is DAPI counterstaining for cell nuclei. (B) Myofibroblasts were isolated from adult human ileostomy surgical samples and plated for four days prior to immunofluorescence staining. Similar to murine myofibroblasts, SMA and vimentin stains were positive while the desmin stain was negative. (C) Myofibroblasts were isolated from a human infant ileostomy and plated for four days prior to immunofluorescence staining. Like the adult human sample, SMA was positive but faint. Vimentin stains were positive while the desmin stain was negative. (D) PCR results performed on the C57 BL/6 murine myofibroblasts consistent with the immunofluorescence results of positive SMA and vimentin staining and negative desmin staining.

In order to identify the ISEMFs obtained from C57BL/6 mouse small intestine, immunofluorescence was used to confirm characteristic specific markers for myofibroblasts. Cells stained positive for α smooth muscle actin (SMA) and vimentin and negative for desmin (Fig. 1A). Quantitative real-time PCR was used to examine the mRNA expression of SMA, vimentin, and desmin (Fig. 1D). Adult and infant human small intestinal samples were stained and demonstrated characteristic myofibroblast staining. However, the SMA staining was not as uniformly strong as that found in the murine myofibroblasts (Figs. 1B and 1C). Quantitative real-time PCR of mRNA from human myofibroblasts yielded a similar pattern of SMA, vimentin, and desmin expression as the murine myofibroblasts.

### Human Intestinal Crypts *in vitro* with Respect to Time

**Figure 2:**
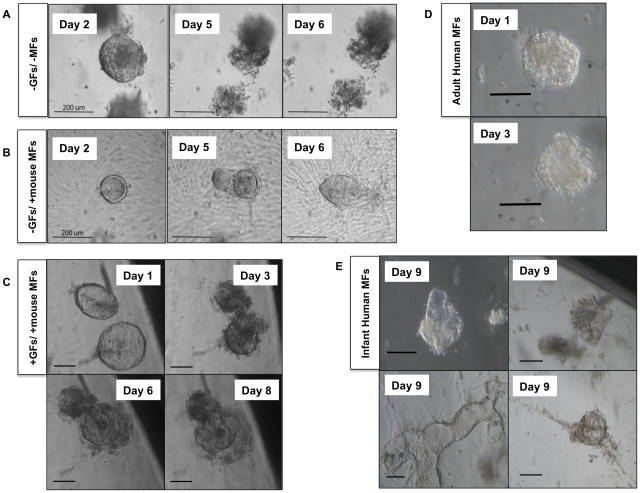
Supportive Effect of Wnt, FGF Growth Factors and Myofibroblasts on Human Epithelial Growth. (A) Human small intestinal crypts cultured without myofibroblasts (MFs) or growth factors (GFs) will live for approximately two days before dying off. (B) Human small intestinal crypts cultured in the presence of murine myofibroblast but without growth factors maintain their cystic shape indefinitely but without significant growth. (C) Human small intestinal crypts cultured with Wnt3a and FGF10 growth factors in the presence of mouse myofibroblasts began as simple cysts that fill and extruded their contents and became more complex in morphology over time. (D) Human epithelial clusters grown on adult human myofibroblasts in the presence of growth factors organize into simple cysts but cannot be maintained for longer than 3 days. (E) Infant human myofibroblasts are capable of supporting human intestinal epithelial growth into complex cystic structures through 9 days post-explantation. For A and B, scale bar is 200 µm. For C, D, and E, scale bar is 100 µm.

Human crypts were isolated from small intestinal surgical samples, suspended in Matrigel and placed into 24-well plates without a myofibroblast feeder layer (n = 16). Intestinal spheroids were observed until culture day 2 but by day 3 their distinct epithelial borders broke down and the spheroids rendered non-viable (Fig 2A). However, when human crypts were placed upon a murine myofibroblasts feeder layer, the epithelial cells were sustained for at least 56 days *in vitro*, maintaining their distinct borders (n = 16). Such cultures were observed on a daily basis during the first week, and on a weekly basis subsequently. Their morphology remained essentially that of a simple cyst without complex structures throughout their growth period (Fig. 2B). Such cystic structures were observed approximately 80% of the time when grown on murine myofibroblasts.

The effect of growth factors Wnt and FGF on epithelial development was evaluated using human crypts grown on murine myofibroblasts. Serial images were obtained of the cultured intestinal crypts over time for 8 days. Isolated crypts quickly developed into simple spheroids with well-defined borders (Fig. 2C, day 1). Over the next day these spheroids filled with debris from the epithelial lining that was eventually extruded from the lumen of the enterospheres (Fig. 2C, day 3). Over the next several days (Fig. 2C, to day 8), there was additional growth of the enterospheres and their morphology became more complex with increasing folding of the cyst wall, which will be called ‘enteroids.’ Most of the viable enteroids were found in the periphery of the culture wells, a common finding with murine enteroids culture models (data not shown, [15]).

**Figure 3:**
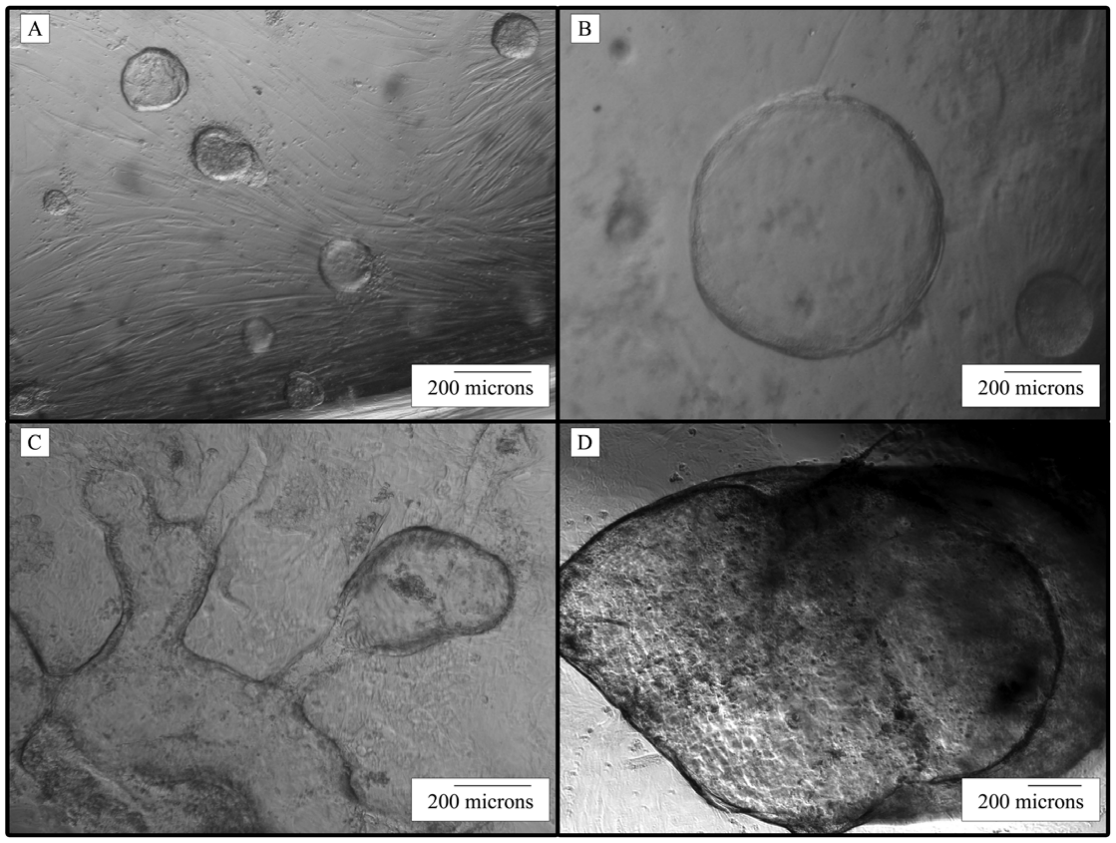
Long-Term Culture of Human Small Intestinal Enteroids Grown on Human Infant Myofibroblasts. Micrographs of human small intestinal crypts that were cultured on human infant myofibroblasts for (A) 2 days, (B) 8 days, (C) 23 days, and (D) 56 days in culture. Small cysts get larger with time and sometimes fuse with each other to form larger structures.

Adult human myofibroblasts were isolated from small intestinal surgical samples, plated, and used to assess their ability to support isolated human intestinal crypts. These enterospheres that were cultured in the presence of these adult human ISEMFs remained viable for 2–3 days (n = 9). They then promptly lost their distinct edge and became non-viable (Fig. 2D, and data not shown). However, we were able to grow adult human enteroids for longer time periods on myofibroblasts isolated from a human infant (n = 8, Fig. 2E). Such enteroids formed every time, and they remained viable for more than 56 days (Figure 3). Interestingly, the enteroids grown on human infant ISEMFs did not require the presence of FGF10, Wnt3a or even R-spondin to sustain growth. Enteroids grown in these cultures demonstrated a variety of different morphologies. These morphologies included simple cyst-like structures with thin epithelial walls, cyst-like structures with budding outgrowths, and elongated thin-walled formations (Fig 2E). The size of the cyst-like structures continued to expand, increasing from the 0.2 mm in diameter initially to over 2 mm in the linear dimension (Figure 3D). When the cyst-like structures were small, we were able to transfer them into a new culture with myofibroblasts, and these structures will continue to expand in size.

### Human Epithelium Expanded in Culture

**Figure 4:**
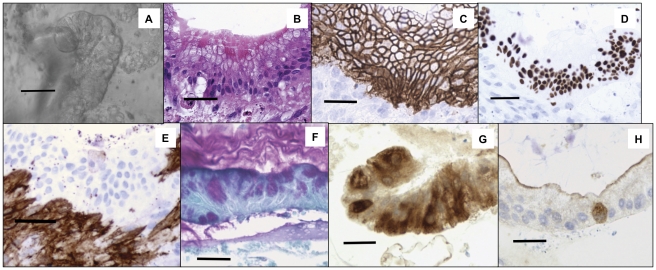
*In vitro* Human Small Intestinal Enteroids Grown on Murine Myofibroblasts Demonstrate Characteristic Intestinal Epithelial Markers. Human crypts cultured on murine myofibroblasts in the presence of Wnt3a and FGF10 were processed after 18 days *in vitro*. (A) Phase contrast microscopy of culture. (B) Hematoxylin and Eosin stain. Note cellular polarity with epithelial nuclei at the basal region and goblet cells at the apical region. (C) E-cadherin, an epithelial cell marker. (D) CDX-2, stains intestinal epithelium. Of note, while the E-cadherin staining is relatively even, the CDX-2 staining demonstrates uneven staining suggestive of alternating crypt-villus domains. (E) Smooth Muscle Actin, marker for myofibroblasts. (F) PAS, stains for goblet cells. Note the extruded mucinous material at the apical side of the epithelium culture. (G) Lysozyme, a Paneth cell marker. (H) Synaptophysin, marker for enteroendocrine cells. For all images, scale bars are 100 µm.

**Figure 5:**
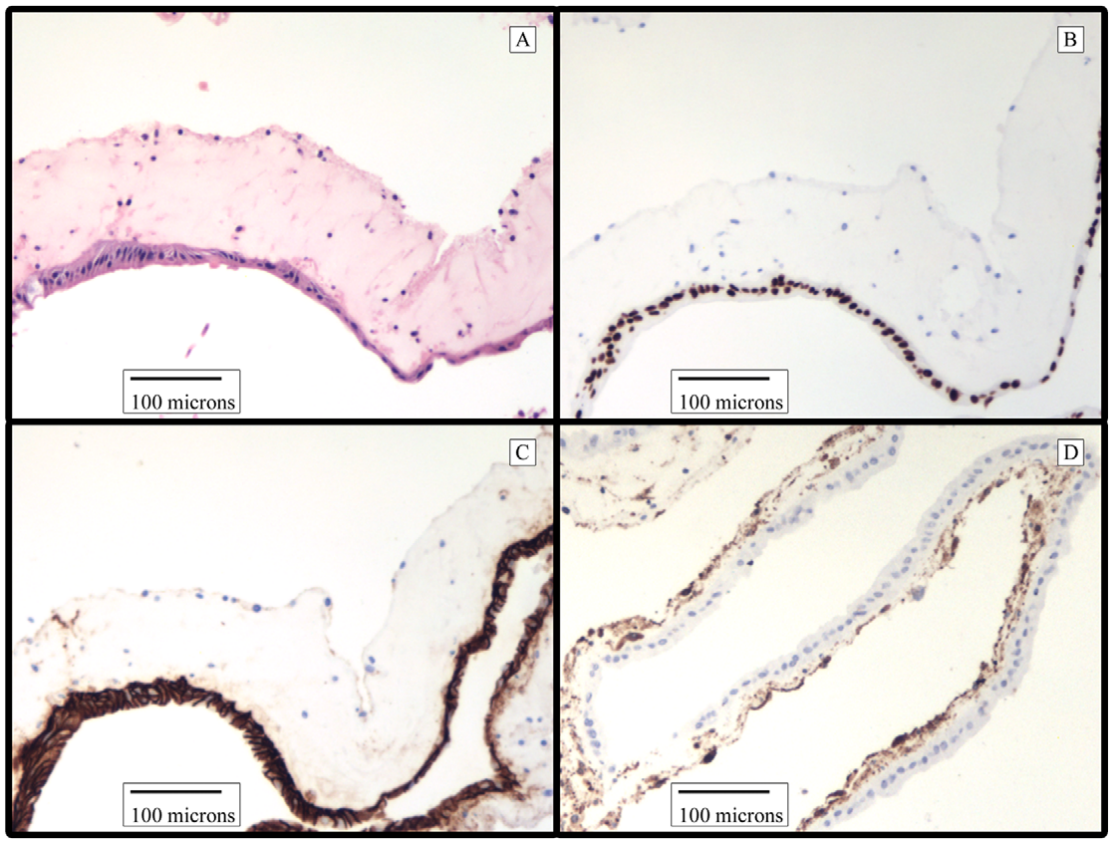
Long-term *In vitro* Human Small Intestinal Enteroids Grown on Murine Myofibroblasts. Human small intestinal crypts cultured on murine myofibroblasts in the presence of Wnt3a and FGF10 were processed after 58 days *in vitro*. (A) Hematoxylin and Eosin stain. (B) CDX-2, stains intestinal epithelium. (C) E-cadherin, an epithelial cell marker. (D) α Smooth Muscle Actin, marker for myofibroblasts.

Some of the human enteroid preparations on murine ISEMFs were allowed to grow until day 18 when they were removed from culture mechanically and processed. Surface markers characteristic for the various cell types of the small intestinal epithelium were used to examine the sections. Phase contrast microscopy of the human epithelial enteroids demonstrated significantly increased complexity of the structures at day 18 as compared to days 1–8 (Fig. 4A). Hematoxylin and Eosin (H&E) staining demonstrated a polarity to the organization of the formed epithelium (Fig. 4B). Goblet cells and enterocytes were found at the apical pole while nuclear staining was found in the basal region of the enteroid. Immunohistochemical stains on the cultures with E-cadherin and CDX-2 corroborated the intestinal epithelial nature of the cultured cells (Fig. 4C, D). CDX-2 is typically expressed more intensely in the crypt base of the intestinal epithelium. The intensity of the nuclear staining by CDX-2 was irregular, suggestive of separate crypt and villus domains within the enteroid. Myofibroblasts were positive for SMA and were found in abundance at the basal side of the enteroid (Fig. 4E). Goblet cells were evaluated using Periodic-acid Schiff staining. Periodic-acid Schiff positive cells were present in the epithelium with extruded mucoid material present in the adjacent overlying region (Fig. 4F). Typically Paneth cells are morphologically pyramidal shaped and lysozyme-positive and are located in the crypt bases between crypt base columnar cells [15]. We demonstrated alternating dark lysozyme positive staining of pyramidal cells in culture (Fig. 4G). Enteroendocrine cells are rare cells in the intestinal epithelium and stain positively with Synaptophysin and Chromogranin A [16]. Here we demonstrate the presence of enteroendocrine cells using Synaptophysin immunohistochemical staining (Fig. 4H). When human epithelial crypts were grown on murine ISEMFs for 58 days, H&E staining demonstrated a polarized epithelial layer (Fig 5A). These epithelial cells were CDX-2 and E-cadherin positive (Fig 5B & C, respectively). In contrast, the adjacent myofibroblasts were positive for SMA (Fig 5D).

### Human Intestinal Epithelium Implanted Subcutaneously in Immunocompromised Mice

**Figure 6:**
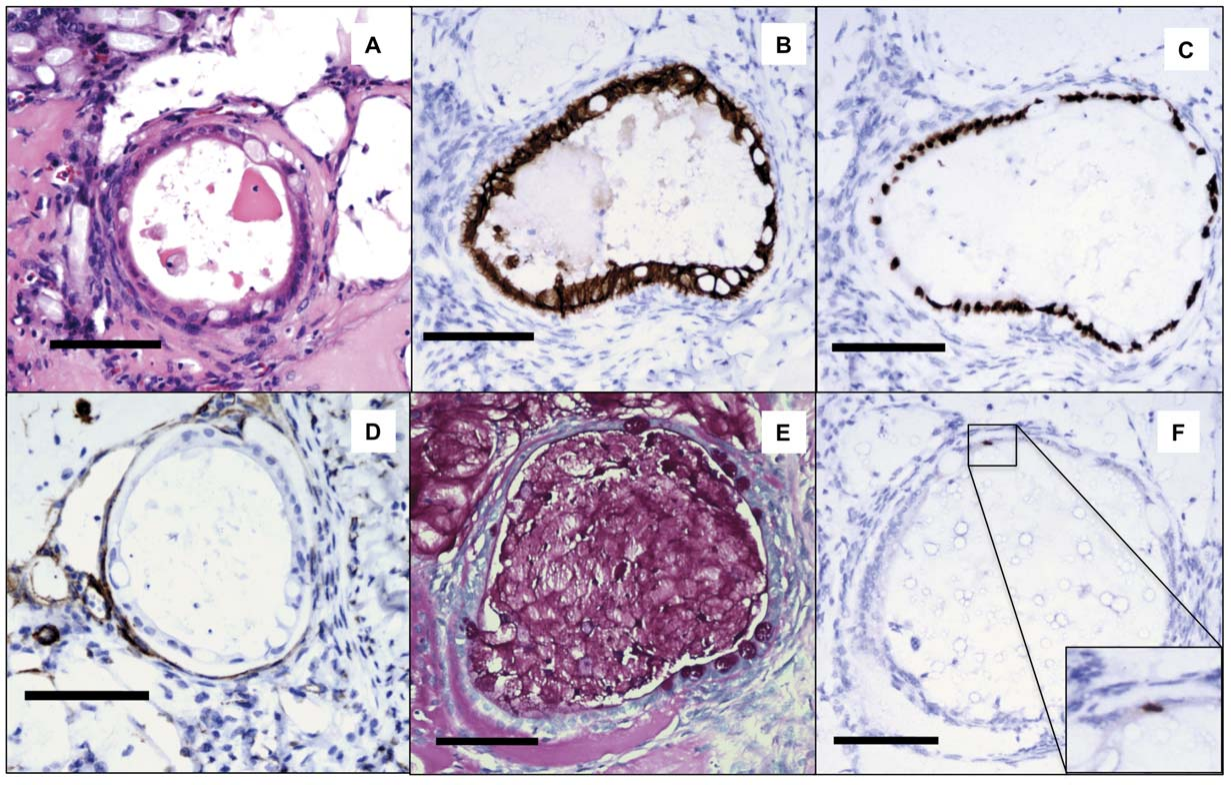
*In vivo* Human Small Intestinal Enteroids Can Be Maintained with Murine ISEMFs. Human small intestinal crypts on murine myofibroblasts were grown in culture for 11 days in the presence of Wnt3a and FGF10, and then placed on a PGA felt scaffold and implanted subcutaneously into an immunocompromised NOD-SCID-IL2Rγ null mice. After 28 days, the implant was harvested and evaluated with intestinal epithelial markers. (A) H&E demonstrates at least three cell morphologies, and eosinophilic material in the cyst lumen. (B) E-cadherin and (C) CDX-2, are intestinal epithelial cell markers. Again note the variable staining intensity by the CDX-2 suggestive of crypt and villus domains (D) α Smooth Muscle Actin staining for myofibroblast adjacent to epithelial cells. (E) PAS staining for mucin and mucin producing goblet cells (F) Chromogranin A, marker for enteroendocrine cells. For all images, scale bar is 100 µm.

**Figure 7:**
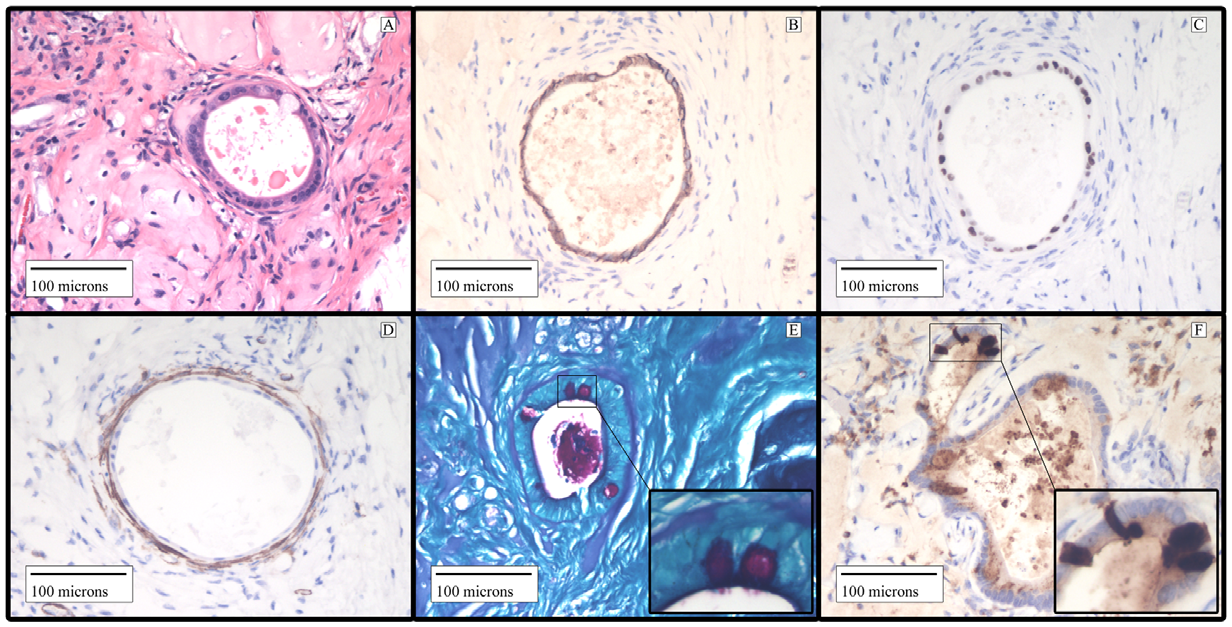
*In vivo* Human Small Intestinal Enteroids Can Be Maintained with Human Infant ISEMFs. Human small intestinal crypts were grown on human myofibroblasts for 8 days, and then placed on a PGA felt scaffold and implanted subcutaneously into an immunocompromised NOD-SCID-IL2Rγ null mice. After 28 days, the implant was harvested and evaluated with intestinal epithelial markers. (A) H&E staining showing epithelial organization. (B) E-cadherin and (C) CDX-2 are intestinal epithelial cell markers. (D) α Smooth Muscle Actin staining for myofibroblast surrounding epithelial cells. (E) PAS staining for mucin and mucin producing goblet cells. (F) Lysozyme, marker for Paneth cells.

Human epithelial enteroids and associated murine ISEMF were cultured for 11 days before being placed upon polyglycolic acid (PGA) scaffolds that were implanted subcutaneously in NOD-SCID-IL2Rγ null (NSG) mice (n = 2). The time span of 11 days was selected due to the ISEMFs and their epithelial contents detaching from the culture well at that point, and thereby facilitating placement on the polyglycolic scaffold. The scaffolds were harvested after 28 days to ascertain their contents' viability and intestinal marker expression. The implantation period of 28 days was chosen because no epithelial culture could be maintained without growth factor support for this length of time in our previous experience. Once excised, the implants were 10% formalin fixed and stained. H&E demonstrated epithelial cysts with eosinophilic luminal contents and with at least three morphologically different cell types (Fig. 6A). Similar to the epithelial cultures, E-cadherin and CDX-2 were positive in the cell lining in the cyst wall, indicating the presence of intestinal epithelium (Fig. 6B, C). The CDX-2 stain again demonstrated domains of irregular staining suggestive of crypt and villus domains along the epithelial cyst. SMA-positive cells suggestive of myofibroblasts were seen in close association with the cyst, surrounding the lining cells of the cyst (Fig. 6D). PAS staining again demonstrated the presence of mucin-producing cells consistent with goblet cells (Fig. 6E). The lumen of the cyst also stained strongly for mucin. Like synaptophysin, chromogranin A also stains enteroendocrine cells. Consistently, multiple stains of the intestinal clusters demonstrated isolated chromogranin A staining. This particular epithelial cyst demonstrated a single cell positive for chromogranin A (Fig. 6F). When human epithelial enteroids cultured with human infant ISEMF were implanted in the same animal model, cysts analogous to those cultured with murine ISEMF also formed (Figure 7). Immunohistochemical staining confirmed the expression of CDX-2 and E-cadherin in the epithelial cells, as well as the expression of SMA in cells surrounding the cysts. Mucin and lysozyme were also present in some of the cells, indicating the presence of goblet and Paneth cells, respectively. Attempts to implant epithelial cells without first establishing this culture with ISEMF led to no growth of epithelium in this model.

## Discussion

The intimate contact between the small intestinal epithelium and the associated sub-epithelial myofibroblasts in both mouse and humans is generally thought to facilitate cross talk between the cell types and help to promote the growth and differentiation of the overlying epithelium [5], [10]. Here we showed that mouse and human ISEMFs would support the growth of isolated intestinal epithelial̀ cells. In the presence of mouse ISEMFs, human epithelial cell cultures are maintained for at least 60 days. On the other hand, epithelial cultures lacking an ISEMF feeder layer died after only 2–3 days. Isolated mouse ISEMFs maintained ectopically placed human intestinal epithelium *in vivo*, allowing them to survive for 28 days subcutaneously in an immunodeficient mouse model without additional external growth factor support. While there have been descriptions of long-term human intestinal epithelial culture systems previously, all have required transformed cells[17]. This is the first report of maintaining non-neoplastic human intestinal epithelium *in vivo* in an immunocompromised mouse for a prolonged period of time. The intestinal enteroids were suspended in Matrigel, a proprietary proteinacious mixture that contains many of the elements and factors in the extracellular environment. Without support from ISEMFs, these suspended epithelial cells in Matrigel only survive approximately 2 days. As we demonstrated, the human epithelial cells were maintained for longer periods of time in the presence of ISEMFs. These findings are consistent with multiple animal studies concluding that apoptosis is prevented with immediate interaction of the epithelium with some matrix element while longer term culture of epithelium *in vitro* requires some mesenchymal element for support[12].

Another interesting finding was our observation that enteroids develop apparent villus and crypt domains in formations of otherwise morphologically similar cells, both in the culture and *in vivo* specimens. This phenomenon was seen most clearly with the CDX-2 staining variability. The finding is suggestive that even within a week of epithelial growth, there is organizational behavior at the cellular level. FGF4 and Wnt3a have been implicated previously in determining the cellular fate of human pluripotent stem cells into hindgut specific domains [18]. It was not until Wnt and FGF were added to our culture systems that the human epithelial cysts became more complex in morphology and dramatically expanded in size. Further understanding the role that other growth factors have will hopefully increase yields of surviving epithelium and improve their growth.

The cues that allow human ISEMFs to support human intestinal enteroids remain to be determined. We were able to support human epithelial growth *in vitro* for at least 60 days using human myofibroblasts. This supportive interaction proved significantly more difficult to demonstrate experimentally than had been the case with the mouse myofibroblasts. We had tested the ability of adult human myofibroblasts' to support the intestinal epithelium but were unsuccessful. In contrast, ISEMF cells isolated from infant human small intestine supported the long-term growth of the epithelial cell cultures. The ‘classic’ myofibroblasts markers in the cells were similar and consistent with those of the mouse. Clearly other age-dependent factors contribute to the long-term support of the human epithelium. These remain to be elucidated. Myofibroblasts are likely one of a number of heterogeneous mesenchymal cells in the lamina propria that have various roles in supporting intestinal epithelium. Likely some of the roles attributed to the ISEMFs may actually be due to other SMA-positive cells[12]. Further assessments of the subtle factors that make the various myofibroblast lines different are certainly warranted.

Our findings lend themselves to further experiments aimed at clarifying the specific factors by which the ISEMFs support the epithelium. By understanding these interactions, it may, for example, be possible to expand single intestinal stem cells into viable human intestinal epithelium using human ISEMF cells as a supportive cell layer. Additionally, the relationship between the crypt stem cells, Paneth cells, and ISEMFs will also need to be investigated to further delineate the molecular interaction between these cell types. Maintenance of these cultures in an *in vivo* environment opens possibilities of long-term cell viability without continuous external supplementation of cytokines and growth factors.

## Materials and Methods

### Ethics Statement

For human tissues, fresh tissues were obtained with appropriate IRB approval from the UCLA Department of Pathology Translational Pathology Core Laboratory.

All animal studies were approved by the animal research committee at UCLA, IRB #2005-169. The UCLA facility is an AALAC-accredited facility. This study was carried out in strict accordance with the recommendations in the Guide for the Care and Use of Laboratory Animals of the National Institutes of Health. All efforts were made to minimize suffering.

### Mouse Care

Myofibroblasts were isolated from 5-day-old C57BL/6 wild type mice from our own breeding colony. Six-week-old immunocompromised NOD-SCID-IL2Rγnull (NSG) mice (Jackson Laboratory, Bar Harbor, Maine) were used for human epithelial implantation studies. Both strains of mouse were housed in the UCLA animal facility. The mouse pups were sacrificed per UCLA Division of Laboratory Animal Medicine (DLAM) protocol using an isoflurane overdose followed by decapitation. NSG mice were placed into a CO2 chamber and gas added per DLAM protocol. The UCLA facility is an AALAC-accredited facility. This study was carried out in strict accordance with the recommendations in the Guide for the Care and Use of Laboratory Animals of the National Institutes of Health. All efforts were made to minimize suffering.

### Human Intestinal Tissue

Non-diseased small intestinal samples were obtained fresh from intestinal specimens excised for Roux-en-Y gastric bypass procedures and uncomplicated ileostomy takedown procedures. Samples were obtained from the Surgical Pathology Department within 45 minutes of resection and placed into ice-cold Dulbecco's Phosphate Buffered Solution (PBS). Fresh tissues obtained with appropriate IRB approval from the UCLA Department of Pathology Translational Pathology Core Laboratory.

### Isolation of intestinal crypts

Tissue was removed from PBS solution and washed multiple times with ice-cold PBS washes until the solution remained clear. The specimen was then placed in a Petri dish containing PBS on ice with the mucosal surface facing upward. Using a razor blade, excess mucoid material was scrapped from the epithelial surface. The specimen was then divided into approximately 0.5 cm^2^ pieces. These pieces were placed into a 2.5 mmol/L EDTA solution in PBS for 30 minutes of incubation with gentle shaking at 4°C. After this incubation period, the fragments were allowed to settle and the supernatant was discarded. 10 ml of cold PBS was added to the sample, and subsequently vortexed for 10 seconds with 1-second bursts. The fragments were allowed to settle, and the supernatant was removed and saved on ice. Again 10 ml of PBS were added and the process was repeated eight times. Samples were spun down at 100 g for 2 minutes. The supernatant was discarded. The contents of the pellets were examined under light microscopy using a Nikon TMS microscope to assess purity of crypt fractions. Typically, all fractions were pooled together to increase yield of epithelial crypts. The pooled fractions were then purified using a 100-µm pore filter (BD Biosciences, Bedford, MA). Fetal Bovine Serum at 10% per volume was then used to suspend the contents of the filtrate. These clusters were examined under light microscopy and counted. 500 crypt clusters were suspended in 50 µL Matrigel (BD Biosciences) as previously described in Sato's 3-D Matrigel culture system developed for murine intestines[15]. The crypt cell/Matrigel suspension was placed directly upon previously plated mouse/human myofibroblasts. Matrigel was allowed to polymerize on the myofibroblasts. Crypt culture medium was then added to the wells. The media consisted of Advanced DMEM/F12 (Invitrogen, Carlsbad, CA) with penicillin-streptomycin (Invitrogen), GlutaMax supplement (Invitrogen, 2 mmol/L), HEPES buffer (Invitrogen, 10 mmol/L), N-2 supplement (Invitrogen), B-27 supplement (Invitrogen), EGF (PeproTech, Rocky Hill, NJ, 50 µg/mL), Murine noggin (PeproTech, 100 µg/mL) and R-spondin (R&D Systems, Minneapolis, MN, 1 µg/mL)[15]. Subsets of studies utilized various doses of Wnt3a (R&D Systems, 100 ng/mL), and FGF10 (R&D Systems, 100 ng/mL). The medium was replaced every two days with the same factors.

### Myofibroblast isolation and culture

Small intestine was excised from 7 day-old mice. The tissue was placed into a Petri dish containing calcium and magnesium free Hank's Buffered Salt Solution (Invitrogen) with D-Glucose (Sigma, 20 mg/mL), penicillin-streptomycin (Invitrogen), and L-glutamine (Invitrogen, 4 mmol/L) (HBSS* solution). The intestines were washed out and rinsed. The intestinal tissue was diced into 0.3–0.5 mm^2^ pieces. The diced material was transferred into a T25 flask. 30 mL of cold HBSS* solution was added to the flask after which the flask was shaken for 2 minutes at room temperature. The flask was then allowed to settle and the supernatant discarded. This process was repeated until the solution was clear.

Once the last supernatant was discarded, a 20 mL dispase (Invitrogen, 0.31 mg/mL)/collagenase Type XI (Sigma, St. Louis, MO, 0.25 mg/mL) solution was added to the tissue. The flask was gently rocked moderately for 30 minutes at room temperature.

The flask contents were then transferred to a 50 mL conical tube and vigorously shaken for 30 seconds. 10 mL cold HBSS* was added to the solution and entire contents allowed to settle. The supernatant was transferred to a new 50 mL conical tube. This was repeated 6 times in 50 ml conical tubes.

The samples were then suspended in 25 mL of high glucose Dulbecco's Modified Eagle Medium with fetal bovine serum (Invitrogen, 5% v/v), L-glutamine (Invitrogen, 4 mmol/L), D-Sorbitol (Sigma, 20 mg/mL), and penicillin-streptomycin (Invitrogen) (DMEM-S solution). The solution was inverted until well mixed and then centrifuged at 100 g at 4°C for two minutes. The tube was then placed back on ice and the supernatant discarded. The pellet was transferred to a 5 mL centrifuge tube. The contents were allowed to settle and any supernatant was discarded. The pellet was resuspended in HBSS with magnesium and calcium supplemented with penicillin-streptomycin (Invitrogen) and L-glutamine (Invitrogen, 4 mmol/L). The entire contents were spun at high speed for 10 seconds. The supernatant was discarded and the pellet was suspended in Basic Growth Media for myofibroblasts. Basic Growth Media consisted of DMEM (Invitrogen), with Antibiotic-Antimycotic (Invitrogen), fetal bovine serum (Invitrogen, 10% v/v), EGF (PeproTech, Rocky Hill, NJ, 50 µg/mL), transferrin (Sigma, St. Louis, MO, 10 µg/mL), and insulin (Sigma, St. Louis, MO, 0.25 U/mL) added.

### Immunohistochemistry

Immunohistochemical studies were undertaken using paraffin-embedded culture samples that were prepared as follows: culture samples were washed once with PBS. The samples were then fixed for ∼12 hours with 10% buffered formalin solution. The formalin solution was then removed and 80% ethanol solution added for 10 minutes then removed. A 95% ethanol solution was added for 15 minutes twice. Finally, 100% ethanol was then added for 10 minutes. The culture contents were then carefully removed mechanically from the culture dish. The samples were then paraffin embedded. Serial 8 µm cuts of the tissue were obtained for microscopic evaluation and staining. Immunohistochemical staining was performed using the DAKO (Carpinteria, CA) automated Flex system. Primary antibodies CDX-2, E-cadherin, SMA, Synaptophysin were obtained from DAKO and were at manufacturer concentrations. Antibody to lysozyme (DAKO) was diluted 1∶1500 in Antibody Diluent (DAKO). Antibody to Chromogranin A (Immunostar, Hudson, WI) was diluted 1∶200 in Antibody Diluent (DAKO).

### Implantation

7–11 days old culture samples were allowed to elevate and partially detach from the culture plate as part of their natural growth process. These samples were placed on non-woven 5 mm polyglycolic acid (PGA) felt disks (Synthecon, Houston, TX). Immunocompromised NOD-SCID-IL2Rγ null (NSG) mice were anesthetized in a manner consistent with protocols establish by the UCLA DLAM group (http://www.ncbi.nlm.nih.gov/pubmed/19052619). A subcutaneous pocket was created in the anterior abdominal wall. The PGA felt with the cultured cells was placed into the pocket and 6–0 Prolene suture (Ethicon, Somerville, NJ) was used to suture the scaffold to underlying muscle. The overlying incision was closed without tension using 3–0 silk (Ethicon, Somerville, NJ) suture. The mouse was sacrificed after 28 days and the implantation was excised and fixed in buffered 10% formalin solution. The sample was embedded, sectioned, and stained for microscopic evaluation.

### Quantitative real-time PCR

mRNA was isolated from the samples with the RNeasy RNA Isolation Kit (Qiagen, Valencia, CA) following the manufacturer's protocol. The mRNA samples were then prepared for the RT-PCR reaction with the Quantitect Probe RT-PCR Kit (Qiagen) and the TaqMan Gene Expression Assay (Applied Biosystems, Carlsbad, CA) for smooth muscle actin (Assay ID Mm01546133_m1), desmin (Mm00802455_m1), vimentin (Mm00449208_m1), and GAPDH (Mm99999915_g1). GAPDH was used as the house keeping gene to normalize RNA quantities. The samples were analyzed with the LightCycler 480 Real-Time PCR System (Roche, Indianapolis, IN) with settings described in the Quantitect Probe Kit. The comparative C^T^ method was used to calculate the relative gene expression.
